# Percutaneous Electrolysis, Percutaneous Peripheral Nerve Stimulation, and Eccentric Exercise for Shoulder Pain and Functionality in Supraspinatus Tendinopathy: A Single-Blind Randomized Clinical Trial

**DOI:** 10.3390/jfmk10030295

**Published:** 2025-07-30

**Authors:** Jorge Góngora-Rodríguez, Manuel Rodríguez-Huguet, Daniel Rodríguez-Almagro, Rocío Martín-Valero, Pablo Góngora-Rodríguez, Carmen Ayala-Martínez, Miguel Ángel Rosety-Rodríguez

**Affiliations:** 1 Department of Nursing and Physiotherapy, University of Cádiz, 11009 Cádiz, Spain; jorge.gongora@uca.es; 2 Department of Nursing, Physiotherapy and Medicine, University of Almería, 04120 Almería, Spain; dra243@ual.es; 3 Department of Physiotherapy, Faculty of Health Sciences, University of Málaga, 29071 Málaga, Spain; rovalemas@uma.es; 4 Doctoral School, University of Cádiz, 11003 Cádiz, Spain; pablo.gongorarodriguez@alum.uca.es (P.G.-R.); carmenmaria.ayamar@alum.uca.es (C.A.-M.); 5 Move-It Research Group, Department of Physical Education, Faculty of Education Sciences, University of Cádiz, 11519 Puerto Real, Spain; miguelangel.rosety@uca.es; 6 Biomedical Research and Innovation Institute of Cadiz, Puerta del Mar University Hospital, University of Cádiz, Plaza Fragela, s/n, 11003 Cádiz, Spain

**Keywords:** eccentric exercise, percutaneous electrolysis, peripheral nerve stimulation, physical therapy, rehabilitation, supraspinatus tendinopathy

## Abstract

**Objectives**: This study aimed to investigate the efficacy of Percutaneous Electrolysis (PE), Percutaneous peripheral Nerve Stimulation (PNS), and Eccentric Exercise (EE) in patients with supraspinatus tendinopathy. **Methods**: Forty-six participants with supraspinatus tendinopathy were randomly allocated to either an invasive therapy group (four sessions in four weeks of PE+PNS and EE program) or a conventional physical therapy group (ten sessions for 2 weeks). The multimodal physical program included Ultrasound therapy (US), Transcutaneous Electric Nerve Stimulation (TENS) and the same EE program. The Numerical Pain Rating Scale (NPRS), shoulder Range of Motion (ROM), Pressure Pain Threshold (PPT), and disability (DASH and SPADI) were measured at baseline, at the end of treatment, and at 12- and 24-weeks follow-up. **Results**: The PE+PNS+EE group demonstrated consistently greater and statistically significant improvements across nearly all pain, mobility, and functional outcomes at all follow-up points (post-treatment, 12-weeks, and 24-weeks) compared to the TENS+US+EE group, with generally medium to large effect sizes. **Conclusions**: This study concludes that the combined PE+PNS+EE intervention offers safe and effective treatment for supraspinatus tendinopathy, demonstrating statistically significant improvements in pain, mobility, and function compared to conventional electrotherapy.

## 1. Introduction

Tendinopathies represent a prevalent cause of pain, functional limitations, and reduced exercise tolerance, significantly impacting functional independence [1,2]. Particularly prevalent in the shoulder joint complex, where tendon injuries are common, these conditions affect diverse population groups, including athletes, manual laborers, and sedentary individuals [3]. Despite extensive research, the etiopathogenesis of tendinopathies has undergone an evolution in understanding, transitioning from the concept of tendinitis to a continuum model that distinguishes between reactive and degenerative tendinopathy, histologically characterized by degeneration, collagen fiber disorganization, and hypervascularization [4,5,6,7,8,9,10]. In addition to clinical examination, the diagnosis of tendinopathies is now supported by novel imaging techniques such as elastography [11,12,13]. Their high incidence and recurrence underscore their relevance within musculoskeletal disorders [14], taking into account the importance of movement for functional independence and the performance of daily activities.

Physical therapy is fundamental in the management of tendinopathies [14,15,16]. Given the need to optimize clinical outcomes in patients with these conditions, particularly in the rotator cuff, invasive ultrasound-guided techniques such as Percutaneous Electrolysis (PE) and Percutaneous peripheral Nerve Stimulation (PNS) have emerged. PE induces a local electrochemical reaction to break down fibrotic tissue and promote regeneration through collagen synthesis [17,18,19]. On the other hand, PNS aims to modulate the nervous system to reduce pain and restore functionality [20]. Additionally, therapeutic exercise, with an emphasis on Eccentric Exercise (EE), is considered an essential tool for improving tendon tissue resilience and stimulating fibroblast activity [21,22].

In this context, there is a recognized need for a comprehensive evaluation comparing the effectiveness of combining invasive physiotherapy techniques (PE+PNS) with EE versus conventional electrotherapy (Transcutaneous Electric Nerve Stimulation (TENS) and Ultrasound therapy (US)) and EE in patients with supraspinatus tendinopathy. New treatments must be highly effective, which is why electrotherapy treatments that can produce effects on the nervous system and modulate pain are becoming increasingly relevant [23].

Therefore, the objective of this study is to analyze changes in pain (Numerical Pain Rating Scale), shoulder Range of Motion (ROM), Pressure Pain Threshold (PPT), and degree of disability (DASH and SPADI) in patients with supraspinatus tendinopathies when comparing the aforementioned treatments, with the aim of gaining a deeper and more comprehensive understanding of the effects of these interventions on supraspinatus pathology and informing the selection of more effective therapeutic strategies. By incorporating a combination of techniques for treating rotator cuff tendinopathies, this study opens a new avenue of research. The aim is to identify the most effective and clinically translatable comprehensive treatment, targeting both tendinous structure and pain perception.

## 2. Materials and Methods

### 2.1. Study Design

A randomized, single-blind clinical trial was conducted in the city of Cádiz, Spain. Between March 2023 and September 2023. A total of 46 volunteer subjects with supraspinatus tendinopathy who were outpatients at a local clinic (Policlínica Santa María) were recruited. All patients were informed of the nature and objectives of the study. This study conforms to consolidated standards of reporting trials guidelines and reports the required information accordingly (CONSORT guidelines for clinical research). The study was approved by the Ethics Committee of Research of Cádiz (Spain) (registration number 149.21.21) on 6 October 2021 and was conducted in accordance with the Declaration of Helsinki. The protocol was registered at ClinicalTrials.gov (registration code NCT05793918) on 15 March 2023.

### 2.2. Sample Size Calculation

Sample size calculation was performed using Epidat software, version 4.2 (Servicio de Epidemiología de la Dirección Xeral de Saúde Pública da Consellería de Sanidade, Xunta de Galicia, Santiago de Compostela, Spain). The sample size calculation was based on obtaining statistically significant differences of two units on the Numeric Pain Rating Scale (NPRS) for pain assessment, with 80% statistical power and a 95% confidence level [24]. This determined a minimum sample size of 18 individuals per group (36 in total) for a study of this nature. However, to account for potential losses, recruitment was expanded to a total of 46 participants with supraspinatus tendinopathy.

### 2.3. Subjects, Randomization, and Blinding

**Figure 1:**
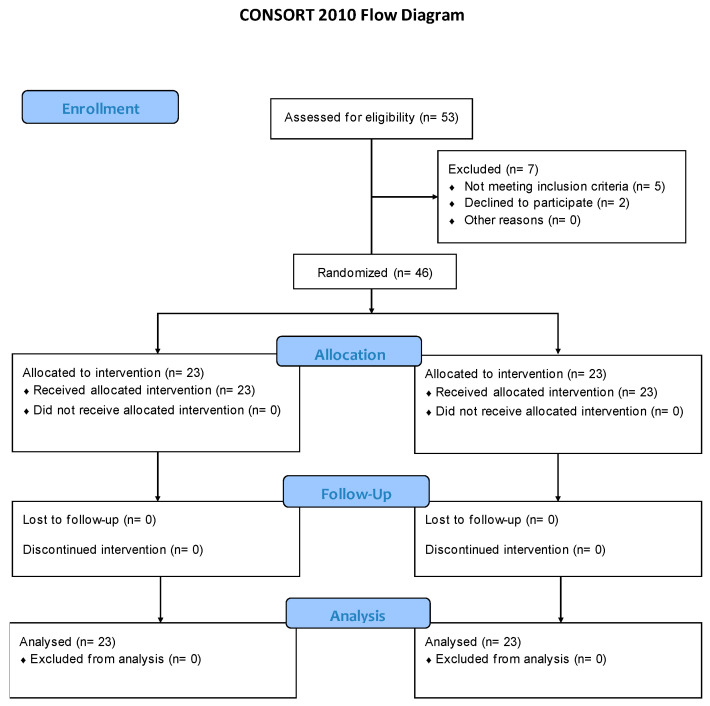
CONSORT flow diagram.

Study recruitment is depicted in Figure 1 following the CONSORT flow diagram. The inclusion criteria were patients of both sexes, aged between 18 and 65 years, diagnosed with supraspinatus tendinopathy (patients had to present with painful symptoms in the sensitive and painful area of the insertion tendon of the supraspinatus muscle in the humerus, reproducible pain on movement against resistance which increased with palpation, and positive results in the Neer, Jobe, and Hawkins–Kennedy tests, without signs of nerve irritation and with no improvement with drug therapy protocols) [25,26]. The exclusion criteria included shoulder pain due to traumatism, fractures, neoplasia, severe osteoporosis, and infectious or inflammatory processes, and patients with pacemakers, congenital anomalies, or pregnancy. Subjects were also excluded if they had received previous PE or PNS treatment in the shoulder joint complex. After receiving comprehensive information regarding the research protocol, each participating patient signed an informed consent form that had been previously approved by the ethics committee.

The randomized sequence for allocation into the treatment groups was created by an independent researcher using a random allocation software program (Epidat 4.2) and was concealed in sequentially numbered envelopes. The randomized sequence was created to generate balanced groups in the number of assigned subjects. Data collection was conducted by a physiotherapist who was blinded as to which participants received a PE+PNS+EE or a TENS+US+EE intervention. After the pretests, 46 participants with supraspinatus tendinopathy were randomly allocated to either the PE group (n = 23) or the GC group (n = 23).

### 2.4. Outcomes Measurements

Pain was the primary outcome variable of the study, assessed using the Numerical Pain Rating Scale (NPRS). This scale measures pain intensity across eleven levels, ranging from 0 (no pain) to 10 (worst possible pain) [24,27]. The NPRS is a validated instrument for pain assessment and demonstrates excellent test–retest reliability. The NPRS showed an intraclass correlation coefficient (ICC) of 0.61 to 0.95, a standard error of measurement (SEM) of 0.48 to 1.02, and a minimum detectable change (MDC) of 1.33 to 2.8 points in patients with musculoskeletal problems. Patients were asked to use the NPRS to rate the pain they were feeling at that moment [28,29].

The degree of disability was specifically analyzed using self-administered questionnaires that correlated the patient’s reported pain with their functional capacity. For this purpose, two well-established instruments were employed. The Disabilities of the Arm, Shoulder, and Hand (DASH) questionnaire served as a specific tool for assessing upper limb function and health-related quality of life in patients with upper extremity pathologies; its Spanish version is a valid and reliable instrument [30]. Furthermore, the Shoulder Pain and Disability Index (SPADI) was utilized to evaluate pain and disability associated with shoulder problems, including rotator cuff pathology [31,32]. The SPADI assesses the patient’s perception of pain and disability in their daily activities [31], and its validated Spanish version is a valuable tool for assessing shoulder pathologies [32].

The Range of Motion (ROM) of the shoulder joint complex was assessed using goniometry with a digital goniometer/inclinometer. The movements evaluated included flexion, extension, abduction, adduction, internal rotation, and external rotation of the shoulder joint complex [25,33].

Pressure Pain Threshold (PPT) was measured using a pressure algometer (Wagner, Baseline FPK, Greenwich, USA), defined as the minimum force applied to induce pain [34,35]. This validated method demonstrates high reliability, with intraclass correlation coefficients exceeding 0.91 [36,37]. The algometer features a 1 cm^2^ rubber disc attached to a pressure pole, and measurements are expressed in kg/cm^2^ with a range of 0 to 10 kg/cm^2^. The evaluator applied the algometer perpendicularly to the skin, increasing pressure incrementally at 1 kg/cm^2^ per second. Patients were instructed to indicate the precise moment the pressure became painful [35]. Measurements were taken at three points on the upper trapezius muscle belly (proximal, middle, and distal), with the distal point approximating the supraspinatus tendon insertion [24].

All follow-up variable measurements were recorded by the same investigator, an experienced physiotherapist who acted as a blinded assessor. The analysis would focus on the patient’s functionality. Although ultrasound was available to guide invasive treatment, and the team specialists had clinical experience, it was considered that ultrasound examination could be evaluator dependent.

### 2.5. Interventions

Participants were divided into two treatment groups. One group received conventional electrotherapy, comprising Transcutaneous Electric Nerve Stimulation (TENS) and Ultrasound therapy (US), while the other group underwent electrotherapy using invasive techniques (PE and PNS). Both groups also participated in a four-week EE program. A highly experienced physiotherapist specializing in shoulder pathology administered the treatments and provided instructions for the exercises.

The TENS and ultrasound (US) treatment was administered five days a week for two weeks. TENS was applied to the affected shoulder using a Megasonic 313 P4 (Electromedicarin^®^, Barcelona, Spain) device. The positive electrode was placed over the supraspinatus muscle and the negative electrode was positioned at the tendinous insertion point. Conventional 5 × 9 cm electrodes were used for 20 min at a frequency of 150 Hz and at a tolerable intensity below the pain threshold, with a pulse duration of 100 µs [38,39].

US was applied for 5 min per session using the 1 cm^2^ transducer of the Megasonic 212 K (Electromedicarin^®^, Barcelona, Spain) device. Treatment parameters included a frequency of 1 MHz and a power of 1.5 W/cm^2^ delivered in continuous mode over the painful area [40,41,42].

The application of PE and PNS techniques required meticulous disinfection of the treatment area, ensuring aseptic conditions throughout [43]. For ultrasound-guided treatments, the ultrasound transducer was covered with a disposable protector in each of the interventions. The intervention spanned four weeks, with one session per week [24,27,44]. PE was applied first, followed by PNS, with both techniques utilizing the EPTE^®^ Bipolar System device (Ionclinics & Deionics S.L., Valencia, Spain). Both interventions were performed under ultrasound guidance to ensure accurate identification of the supraspinatus tendon and the suprascapular nerve using the Mindray^®^ DP30 ultrasound machine (Mindray Bio-Medical Electronics Co., Shenzhen, P.R. China). Acupuncture needles of 0.30 mm thickness and 40 mm length were used [43,44,45].

**Figure 2:**
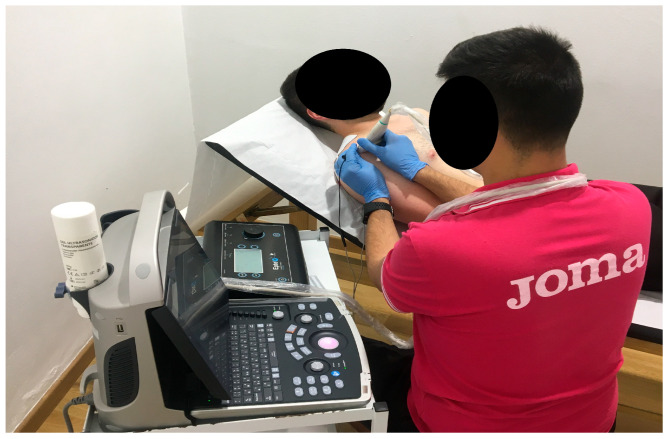
Application of PE treatment.

**Figure 3:**
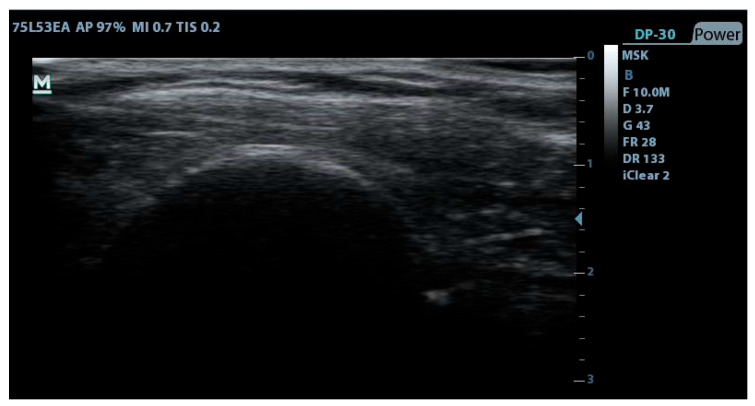
Ultrasound image of PE treatment.

Intratendinous PE treatment (Figure 2) was administered (Figure 3) at an intensity of 350 µA for 72 s during each session. The cathode (needle in the applicator) was positioned along the orientation of the supraspinatus tendon, while the anode (surface electrode) was placed on the upper trapezius muscle. Patients were positioned in supine decubitus with the shoulder in internal rotation for this procedure [24,44,46]. The use of ultrasound allowed for adequate localization of the supraspinatus tendon for treatment. The patients were informed that they could perceive a sensation of paresthesia or burning in the area on the lateral side of the shoulder. The application of the treatment was carried out according to the following description, as represented in the images being performed by a specialized physiotherapist (J.G.R) with 7 years of experience in the use of invasive techniques.

PNS treatment was applied adjacent to the suprascapular nerve within the suprascapular notch, deep to the upper trapezius and supraspinatus muscle. A low-frequency current (10 Hz) was delivered for 90 s at an intensity tolerable to the patient, aiming to elicit a sensory or motor response [20,27].

The eccentric exercise protocol, common to both groups, was designed to be specific, low-intensity, and high-frequency [45,47]. This program included three prescribed exercises performed in 3 sets of 10 repetitions twice daily [48]. The first exercise, performed while standing, focused on supraspinatus activation during shoulder abduction against elastic band resistance, with careful attention to the eccentric return phase. For the second exercise, patients sat with their elbow flexed and supported, performing concentric contraction of the infraspinatus in external rotation and an eccentric return to internal rotation, again using an elastic band for resistance. Finally, subjects worked on global shoulder stability in a quadruped position, moving the shoulder into flexion and executing an eccentric contraction during the return movement [44,45]. The exercises were performed with a high-resistance elastic band. Participants were instructed to focus their attention on the eccentric phase of the exercise and could assist with the unaffected limb in the concentric phase.

### 2.6. Statistical Analysis

Data management and data analysis were performed using the IBM SPSS Statistics package, version 28.0.1.0 (SPSS Inc., Chicago, IL, USA). The level of statistical significance was set at *p* < 0.05. Means and standard deviations were used to describe data for continuous variables and frequencies and percentages were used for categorical variables. Levene’s test and the Kolmogorov–Smirnov test were used to analyze homoscedasticity and normal distribution of continuous variables. Homogeneity between groups at baseline was assessed by one-way analysis of variance (ANOVA) for quantitative variables, and chi-squared test for categorical variables.

A 2 × 4 mixed model repeated measures analysis of variance (ANOVA) was used to analyze the effect of therapy. The correlation of interest was the time-by-group interaction. Variables that showed significant baseline differences between groups were analyzed using a repeated measures analysis of covariance (ANCOVA), incorporating the baseline value of the respective variable as a covariate within the model to control for its potential confounding effect over time. Between-group differences at post-treatment, at 12-weeks follow-up, and at 24-weeks follow-up were determined by Student’s t-test for pre-change and post-change scores. A paired samples t-test was used to assess within-group differences along the two time points. Eta squared (η^2^) was calculated to assess the effect sizes (ESs) for time-by-group interactions, and Cohen’s d was selected to assess the ESs for both the between-group analysis and the within-group analysis [49].

## 3. Results

Finally, study assessments were completed by a total of 46 patients. Subjects were randomly divided into two groups of 23 participants. Men represented the 69.6% of the sample, while women represented 30.4%, with a mean age of the total sample of 44.15 years old (SD = 12.26). The sample did not show differences at baseline, except for disability, which were controlled in the analysis to ensure comparability. All morphological and baseline data are shown in Table 1.

The analysis performed to evaluate the effect of the experimental therapy through time showed statistically significant improvements in favor of the PE+PNS+EE group in all study variables, with effect sizes between medium and large and power values between 0.715 and 1.000 (Table 2), except for ROM in adduction movements (F = 1.746; *p* = 0.193).

The between-group analysis at post-treatment showed statistically significant differences in all study variables except in ROM in adduction movements (mean difference = 1.52; *p* = 0.349) (Table 3). Although both groups showed statistically significant improvement over time the PE+PNS+EE group reflected greater improvements than the TENS+US+EE group (Table 3).

At 12-weeks follow-up, although both the PE+PNS+EE and the TENS+US+EE group showed statistically significant improvements for all study variables over time, the PE+PNS+EE group experienced greater enhancements than the TENS+US+EE group in all study variables except for ROM in extension (mean difference = 2.87; *p* = 0.119) and adduction movements (mean difference = 1.87; *p* = 0.258) (Table 4).

Similar results were obtained at 24 weeks follow-up. The PE+PNS+EE group obtained statistically significant improvements over time in all study variables, while TENS+US+EE group also showed statistically significant enhancements over time in all study variables (Table 5). Despite the above, the PE+PNS+EE group experienced greater enhancements than the TENS+US+EE group in all study variables (Table 5).

## 4. Discussion

The results achieved with the PE+PNS+EE intervention for supraspinatus tendinopathies, compared to the conventional electrotherapy protocol (TENS+US+EE), suggest that combining eccentric exercise (EE) with invasive physiotherapy techniques (PE+PNS) may be an effective option for eliciting positive effects on pain, movement, and functional capacity. These findings align with previous research utilizing similar treatments [24,27,44], thereby reaffirming the potential of invasive physiotherapy techniques for tendinopathies [50,51]. These findings offer promising answers and alternatives to the dissatisfaction patients often experience with commonly established treatments in clinical settings [52,53,54]. Moreover, this research aims to address the existing gap in studies on the effects of electrolysis, as highlighted by recent reviews [55,56,57], given the continued need for in-depth investigation into the most appropriate treatment dosage [17,18].

Percutaneous Electrolysis (PE) operates on the principle of applying galvanic current, allowing current control based on electrical charge, which can be modulated by application time and current intensity [43]. It is estimated that low-intensity PE primarily induces a local analgesic effect, while higher intensity settings can elicit local, segmental, and cortical changes, leading to both analgesic and tissue repair effects [58,59]. However, in a direct comparison of both modalities in healthy subjects, pain modulation was found to be independent of treatment dosage [60]. This indicates that very similar outcomes can be achieved with the same electrical charge, regardless of the applied intensity [61,62]. Focused on shoulder tendinopathies, the research supports treatment protocols utilizing low-intensity current regulated in microamperes (µA) [24,44,45]. In animal models, this low-intensity modality may also be associated with a predominantly mechanical effect [63].

Conversely, the operational parameters for Percutaneous Neuromodulation (PNS) on the suprascapular nerve involve short application times, low frequency, and a tolerable intensity capable of generating a sensory or motor response. This aims to inhibit the input of nociceptive stimuli [20,64].

The findings from this clinical trial indicate that the PE+PNS+EE combined treatment significantly reduced pain levels, both in the short and long term, for patients with supraspinatus tendinopathies. While the conventional treatment of TENS+US+EE also yielded improvements, the experimental treatment demonstrated superior outcomes when examining inter-group differences.

Moreover, the intervention incorporating invasive physiotherapy techniques alongside exercise achieved greater clinical success, with effects observed earlier (by the end of the proposed treatment) and sustained over time. Therefore, the observed positive effects of this treatment can be attributed to the synergistic action of all components of the protocol: PE+PNS+EE.

Thus, the action of PE is justified as a therapy capable of initiating a localized inflammatory response in the injured tendon, thereby triggering repair mechanisms [65] and matrix remodeling [66]. These changes are believed to be dependent on pH modification [59] and the activation of the NLRP3 inflammasome, which is involved in tissue damage recognition and ultimately promotes collagen synthesis [67,68]. Notably, these effects are more significant than those obtained with the application of dry needling [24,69].

Furthermore, PNS facilitates pain control and optimizes muscle function [20,64,70]. Given the established relationship between the suprascapular nerve and rotator cuff tendinopathy [71,72,73], this intervention can lead to improved range of motion, better control of the pathology, and enhanced stabilization of the joint complex.

The combined effects of invasive physiotherapy and therapeutic exercise are observable in the short term and are sustained over time. In contrast, changes in the control group, which received conventional electrotherapy and exercise, appeared over a longer period. This distinction might be linked to the specific effects of eccentric exercise, acknowledging its significant role as a mechanical stimulus in inducing analgesia and reversing the pathological cycle [48,74,75].

Therefore, the ability of invasive techniques, combined with the inclusion of strength exercises, is notable for achieving symptomatic improvements in shoulder tendinopathies [76]. The therapeutic exercise program was based on EE as it is the most widely adopted exercise modality for tendinopathies [77]. However, the analgesic effect and clinical benefits are not exclusive to Eccentric Exercises and can extend to other exercise modalities, such as isometric [74] and combined concentric and eccentric contractions [21,78,79,80].

The results indicate that the analgesic effect and changes in the mechanical capacity of the tendon lead to improvements in shoulder Range of Motion. This is particularly relevant as rotator cuff tendinopathies significantly impact the functional capacity of the entire upper limb and an individual’s independence in daily tasks [25,52,81]. Both groups showed statistically significant differences, supporting the proposed treatments and justifying these changes, primarily due to the inclusion of therapeutic exercise in both interventions. However, the inter-group comparison revealed a greater effect of the experimental intervention on mobility levels.

Finally, it is pertinent to discuss the treatment-derived changes observed in the disability scales, as these reflect the combined modifications in pain and movement on functional capacity. The similar trends observed in both the DASH and SPADI scores suggest that both scales may be equally valid for assessing upper limb disability in supraspinatus tendinopathies, given their widespread use [44,45,82].

The main strength of this study lies in the analysis of the effects of exercise when combined with two novel electrotherapy techniques within the context of invasive physiotherapy. Previous studies have shown that combined therapies may enhance the positive outcomes of individual treatment modalities [83,84]. This offers new possibilities for designing therapeutic interventions for supraspinatus tendinopathy by examining their impact on pain and mobility. The changes observed in NPRS, ROM, PPT, DASH, and SPADI represent significant benefits for patients’ functional independence and quality of life. Treatments that reduce pain will enable individuals to perform daily activities effectively and safely, allowing them to return to more demanding professional activities, including sports.

However, several limitations should be noted. Establishing blinded conditions for both patients and physiotherapists applying these treatments is inherently challenging. Additionally, the effective application of PE and PNS treatments relies heavily on the physiotherapist’s knowledge and skill, while adherence to the Eccentric Exercise program is patient dependent. While all outcome assessments were conducted by a blinded, experienced physiotherapist, following standardized protocols, we acknowledge that self-reported pain measures such as the NPRS may be influenced by factors such as floor or ceiling effects and regression to the mean. Nonetheless, baseline NPRS scores were comparable across groups, and the significant improvements observed in multiple independent measures, including Pressure Pain Thresholds, functional outcomes, and Range of Motion, support the robustness of our findings. Furthermore, the combination of treatment techniques limits the ability to ascertain the isolated effect of each intervention.

Future research could focus on comparing different treatment dosages for both invasive techniques and therapeutic exercise and on promoting patient education from a biopsychosocial perspective. It could be fundamental to establish long-term monitoring of an exercise program’s execution to both assess and promote patient adherence to an active treatment.

## 5. Conclusions

The treatment proposed in this research is a safe and effective option for supraspinatus tendinopathies. The intervention combining PE+PNS+EE yields positive results across the NPRS, PPT, ROM, DASH, and SPADI scales, demonstrating statistically significant improvements in the intergroup analysis when compared to conventional electrotherapy and exercise (TENS+US+EE).

## Figures and Tables

**Table 1:** Morphological and clinical characteristics of the sample and between-groups comparison at baseline.

		ALL (46)		PE+PNS+EE GROUP (23)		TENS+US+EE GROUP (23)		
**CATEGORICAL**		**Frequency**	**%**	**Frequency**	**%**	**Frequency**	**%**	***p***
Sex	Male	32	69.60	17	73.90	15	65.20	0.522
	Female	14	30.40	6	26.10	8	34.80	
Affected Hand	Right	31	67.40	15	65.20	16	69.60	0.753
	Left	15	32.60	8	34.80	7	30.40	
Dominant Hand	Right	43	93.50	22	95.70	21	91.30	0.550
	Left	3	6.50	1	4.30	2	8.70	
**CONTINUOUS**		**Mean**	**SD**	**Mean**	**SD**	**Mean**	**SD**	***p***
Age		44.15	12.26	44.39	13.90	43.91	10.69	0.897
Weight		82.70	11.47	84.04	7.60	81.35	14.41	0.433
Height		1.74	0.07	1.74	0.07	1.74	0.08	0.984
BMI		27.44	4.15	27.87	2.83	27.01	5.18	0.488
NPRS		7.24	1.37	7.52	1.31	6.96	1.40	0.164
PPT Proximal		2.63	0.97	2.62	0.92	2.65	1.04	0.905
PPT Medium		2.78	1.01	2.75	1.09	2.81	0.95	0.853
PPT Distal		2.62	0.98	2.60	1.09	2.63	0.89	0.918
Disability (DASH)		54.72	19.86	62.00	16.46	47.43	20.61	0.011
Disability (DASH) %		45.60	16.55	51.67	13.72	39.53	17.17	0.011
Disability (SPADI)		69.26	19.46	70.83	18.17	67.70	20.96	0.591
Disability (SPADI) %		53.28	14.97	54.48	13.98	52.07	16.12	0.591
Flexion ROM		118.09	22.44	115.00	21.94	121.17	22.99	0.357
Extension ROM		25.20	7.26	26.87	8.00	23.52	6.16	0.119
Abduction ROM		99.98	20.86	104.52	22.80	95.43	18.09	0.142
Adduction ROM		26.80	8.22	27.83	9.79	25.78	6.32	0.406
Internal Rotation ROM		56.54	19.29	57.43	22.18	55.65	16.36	0.758
External Rotation ROM		60.76	21.41	60.96	24.83	60.57	17.92	0.951

Abbreviations %—Percentage; *p*—*p*-value; SD—Standard Deviation; BMI—Body Mass Index; DASH—Disabilities of the Arm, Shoulder and Hand; EE—Eccentric Exercise; NPRS—Numerical Pain Rating Scale; PE—Percutaneous Electrolysis; PNS—Percutaneous peripheral Nerve Stimulation; PPT—Pressure Pain Threshold; ROM—Range of Motion; SPADI—Shoulder Pain and Disability Index; TENS—Transcutaneous Electrical Nerve Stimulation; US—Therapeutic Ultrasound.

**Table 2:** Statistical significance, effect sizes, and power of time-by-group interactions from 2 × 4 mixed model repeated measures ANOVA.

VARIABLE	F	*p*	η2	POWER
NPRS	45.787	<0.001 **	0.510	1.000
PPT Proximal	22.232	<0.001 **	0.336	1.000
PPT Medium	22.464	<0.001 **	0.338	1.000
PPT Distal	17.883	<0.001 **	0.289	1.000
Disability (DASH)	78.393	<0.001 **	0.641	1.000
Disability (DASH) %	78.393	<0.001 **	0.641	1.000
Disability (SPADI)	69.063	<0.001 **	0.611	1.000
Disability (SPADI) %	69.063	<0.001 **	0.611	1.000
Flexion ROM	28.185	<0.001 **	0.390	1.000
Extension ROM	4.840	0.017 *	0.099	0.715
Abduction ROM	7.751	0.002 *	0.150	0.894
Adduction ROM	1.746	0.193	0.038	0.273
Internal Rotation ROM	11.042	0.001 *	0.201	0.927
External Rotation ROM	7.504	0.006 *	0.146	0.806

Abbreviations. η2—Eta squared; *p*—*p*-value; DASH—Disabilities of the Arm, Shoulder, and Hand; NPRS—Numerical Pain Rating Scale; PPT—Pressure Pain Threshold; ROM—Range of Motion; SPADI—Shoulder Pain and Disability Index. *—*p*-value < 0.05. **—*p*-value < 0.001.

**Table 3:** Within-group and between-groups differences in post-treatment.

VARIABLE		Post-Treatment		Within-Group Change Score		Effect Size	Between-Groups Change Score		Effect Size
		Mean	SD	Mean Difference	*p*	*d*	Mean Difference	*p*	*d*
NPRS	PE+PNS+EE	1.57	1.67	−5.96	<0.001	−2.996	−3.61	<0.001 **	−2.323
	TENS+US+EE	4.61	1.62	−2.35	<0.001	−2.512			
PPT Proximal	PE+PNS+EE	4.09	0.99	1.47	<0.001	1.947	0.88	<0.001 **	1.323
	TENS+US+EE	3.24	1.01	0.59	<0.001	1.050			
PPT Medium	PE+PNS+EE	4.11	0.93	1.36	<0.001	1.505	0.91	<0.001 **	1.261
	TENS+US+EE	3.25	1.02	0.44	<0.001	0.914			
PPT Distal	PE+PNS+EE	4.06	1.03	1.46	<0.001	1.442	0.89	0.001 *	1.033
	TENS+US+EE	3.20	0.90	0.57	<0.001	0.825			
Disability (DASH)	PE+PNS+EE	11.26	10.99	−50.74	<0.001	−3.007	−36.83	<0.001 **	−2.531
	TENS+US+EE	33.52	24.12	−13.91	<0.001	−1.182			
Disability (DASH) %	PE+PNS+EE	9.38	9.16	−42.28	<0.001	−3.007	−30.69	<0.001 **	−2.531
	TENS+US+EE	27.93	20.10	−11.59	<0.001	−1.182			
Disability (SPADI)	PE+PNS+EE	15.17	12.42	−55.65	<0.001	−3.353	−35.35	<0.001 **	−2.601
	TENS+US+EE	47.39	20.66	−20.30	<0.001	−2.094			
Disability (SPADI) %	PE+PNS+EE	11.67	9.56	−42.81	<0.001	−3.353	−27.19	<0.001 **	−2.601
	TENS+US+EE	36.45	15.89	−15.62	<0.001	−2.094			
Flexion ROM	PE+PNS+EE	155.04	13.49	40.04	<0.001	2.057	22.70	<0.001 **	1.443
	TENS+US+EE	138.52	23.70	17.35	<0.001	1.612			
Extension ROM	PE+PNS+EE	36.65	5.99	9.78	<0.001	1.547	4.04	0.015 *	0.749
	TENS+US+EE	29.26	5.50	5.74	<0.001	1.342			
Abduction ROM	PE+PNS+EE	143.39	17.50	38.87	<0.001	2.232	14.26	0.004 *	0.903
	TENS+US+EE	120.04	23.44	24.61	<0.001	1.760			
Adduction ROM	PE+PNS+EE	32.91	5.40	5.09	0.002	0.736	1.52	0.349	0.279
	TENS+US+EE	29.35	4.82	3.57	<0.001	1.046			
Internal Rotation ROM	PE+PNS+EE	85.70	8.04	28.26	<0.001	1.372	16.83	<0.001 **	1.084
	TENS+US+EE	67.09	15.17	11.43	<0.001	1.503			
External Rotation ROM	PE+PNS+EE	84.04	10.77	23.09	<0.001	1.202	12.70	0.006 *	0.853
	TENS+US+EE	70.96	17.14	10.39	<0.001	1.202			

Abbreviations. d—Cohen’s d; *p*—*p*-value; SD—Standard Deviation; DASH—Disabilities of the Arm, Shoulder, and Hand; EE—Eccentric Exercise; NPRS—Numerical Pain Rating Scale; PE—Percutaneous Electrolysis; PNS—Percutaneous peripheral Nerve Stimulation; PPT—Pressure Pain Threshold; ROM—Range of Motion; SPADI—Shoulder Pain and Disability Index; TENS—Transcutaneous Electrical Nerve Stimulation; US—Therapeutic Ultrasound. *—*p*-value < 0.05. **—*p*-value < 0.001.

**Table 4:** Within-group and between-groups differences at 12 weeks follow-up.

VARIABLE		12 Weeks Follow-Up		Within-Group Change Score		Effect Size	Between-Groups Change Score		Effect Size
		Mean	SD	Mean Difference	*p*	*d*	Mean Difference	*p*	*d*
NPRS	PE+PNS+EE	0.80	1.23	−6.72	<0.001	−4.054	−3.41	<0.001 **	−2.206
	TENS+US+EE	3.65	2.31	−3.30	<0.001	−2.314			
PPT Proximal	PE+PNS+EE	4.50	0.78	1.88	<0.001	2.011	1.20	<0.001 **	1.530
	TENS+US+EE	3.34	0.93	0.69	<0.001	1.170			
PPT Medium	PE+PNS+EE	4.54	0.72	1.79	<0.001	1.723	1.26	<0.001 **	1.528
	TENS+US+EE	3.34	0.98	0.53	<0.001	1.008			
PPT Distal	PE+PNS+EE	4.49	0.81	1.89	<0.001	1.766	1.21	<0.001 **	1.362
	TENS+US+EE	3.31	0.91	0.68	<0.001	1.031			
Disability (DASH)	PE+PNS+EE	4.09	5.25	−57.91	<0.001	−3.589	−40.43	<0.001 **	−2.866
	TENS+US+EE	29.96	23.39	−17.48	<0.001	−1.489			
Disability (DASH) %	PE+PNS+EE	3.41	4.37	−48.26	<0.001	−3.589	−33.70	<0.001 **	−2.866
	TENS+US+EE	24.96	19.50	−14.57	<0.001	−1.489			
Disability (SPADI)	PE+PNS+EE	7.00	7.29	−63.83	<0.001	−3.670	−38.35	<0.001 **	−2.571
	TENS+US+EE	42.22	22.28	−25.48	<0.001	−2.135			
Disability (SPADI) %	PE+PNS+EE	5.38	5.60	−49.10	<0.001	−3.670	−29.50	<0.001 **	−2.571
	TENS+US+EE	32.47	17.14	−19.60	<0.001	−2.135			
Flexion ROM	PE+PNS+EE	157.17	12.67	42.17	<0.001	2.287	24.96	<0.001 **	1.711
	TENS+US+EE	138.39	24.02	17.22	<0.001	1.863			
Extension ROM	PE+PNS+EE	37.09	5.21	10.22	<0.001	1.448	2.87	0.119	0.469
	TENS+US+EE	30.87	5.07	7.35	<0.001	1.471			
Abduction ROM	PE+PNS+EE	145.43	16.57	40.91	<0.001	2.253	12.35	0.015 *	0.748
	TENS+US+EE	124.00	24.14	28.57	<0.001	1.947			
Adduction ROM	PE+PNS+EE	32.83	5.37	5.00	0.002	0.720	1.87	0.258	0.338
	TENS+US+EE	28.91	4.78	3.13	<0.001	0.867			
Internal Rotation ROM	PE+PNS+EE	86.26	7.93	28.83	<0.001	1.376	15.22	0.002 *	0.958
	TENS+US+EE	69.26	16.49	13.61	<0.001	1.685			
External Rotation ROM	PE+PNS+EE	85.70	8.92	24.74	<0.001	1.238	12.35	0.013 *	0.763
	TENS+US+EE	72.96	17.35	12.39	<0.001	1.111			

Abbreviations. d—Cohen’s d; *p*—*p*-value; SD—Standard Deviation; DASH—Disabilities of the Arm, Shoulder, and Hand; EE—Eccentric Exercise; NPRS—Numerical Pain Rating Scale; PE—Percutaneous Electrolysis; PNS—Percutaneous peripheral Nerve Stimulation; PPT—Pressure Pain Threshold; ROM—Range of Motion; SPADI—Shoulder Pain and Disability Index; TENS—Transcutaneous Electrical Nerve Stimulation; US—Therapeutic Ultrasound. *—*p*-value < 0.05. **—*p*-value < 0.001.

**Table 5:** Within-group and between-groups differences at 24 weeks follow-up.

VARIABLE		24 Weeks Follow-Up		Within-Group Change Score			Between-Groups Change Score		Effect Size
		Mean	SD	Mean Difference	*p*	*d*	Mean Difference	*p*	*d*
NPRS	PE+PNS+EE	0.57	0.90	−6.96	<0.001	−4.571	−3.61	<0.001 **	−2.579
	TENS+US+EE	3.61	2.13	−3.35	<0.001	−2.646			
PPT Proximal	PE+PNS+EE	4.75	0.55	2.13	<0.001	2.539	1.34	<0.001 **	1.846
	TENS+US+EE	3.44	0.94	0.79	<0.001	1.319			
PPT Medium	PE+PNS+EE	4.75	0.49	2.00	<0.001	1.989	1.39	<0.001 **	1.72
	TENS+US+EE	3.42	0.99	0.61	<0.001	1.138			
PPT Distal	PE+PNS+EE	4.73	0.60	2.12	<0.001	2.176	1.40	<0.001 **	1.635
	TENS+US+EE	3.36	0.88	0.72	<0.001	1.004			
Disability (DASH)	PE+PNS+EE	1.87	2.58	−60.13	<0.001	−3.694	−41.30	<0.001 **	−2.89
	TENS+US+EE	28.61	23.31	−18.83	<0.001	−1.571			
Disability (DASH) %	PE+PNS+EE	1.56	2.15	−50.11	<0.001	−3.694	−34.42	<0.001 **	−2.89
	TENS+US+EE	23.84	19.43	−15.69	<0.001	−1.571			
Disability (SPADI)	PE+PNS+EE	4.52	5.84	−66.30	<0.001	−3.758	−39.61	<0.001 **	−2.582
	TENS+US+EE	41.00	22.01	−26.70	<0.001	−2.115			
Disability (SPADI) %	PE+PNS+EE	3.48	4.49	−51.00	<0.001	−3.758	−30.47	<0.001 **	−2.582
	TENS+US+EE	31.54	16.93	−20.54	<0.001	−2.115			
Flexion ROM	PE+PNS+EE	156.91	12.18	41.91	<0.001	2.208	26.96	<0.001 **	1.772
	TENS+US+EE	136.13	22.27	14.96	<0.001	1.476			
Extension ROM	PE+PNS+EE	37.87	4.75	11.00	<0.001	1.398	4.96	0.012 *	0.768
	TENS+US+EE	29.57	5.42	6.04	<0.001	1.309			
Abduction ROM	PE+PNS+EE	145.04	14.10	40.52	<0.001	2.096	16.70	0.002 *	0.964
	TENS+US+EE	119.26	22.50	23.83	<0.001	1.584			
Adduction ROM	PE+PNS+EE	33.30	5.23	5.48	0.001	0.773	2.65	0.116	0.473
	TENS+US+EE	28.61	4.47	2.83	<0.001	0.799			
Internal Rotation ROM	PE+PNS+EE	85.87	8.21	28.43	<0.001	1.374	15.26	0.002 *	0.961
	TENS+US+EE	68.83	15.85	13.17	<0.001	1.509			
External Rotation ROM	PE+PNS+EE	85.22	9.71	24.26	<0.001	1.220	13.74	0.005 *	0.875
	TENS+US+EE	71.09	17.45	10.52	<0.001	1.066			

Abbreviations. d—Cohen’s d; *p*—*p*-value; SD—Standard Deviation; DASH—Disabilities of the Arm, Shoulder, and Hand; EE—Eccentric Exercise; NPRS—Numerical Pain Rating Scale; PE—Percutaneous Electrolysis; PNS—Percutaneous peripheral Nerve Stimulation; PPT—Pressure Pain Threshold; ROM—Range of Motion; SPADI—Shoulder Pain and Disability Index; TENS—Transcutaneous Electrical Nerve Stimulation; US—Therapeutic Ultrasound. *—*p*-value < 0.05. **—*p*-value < 0.001.

## Data Availability

All data are contained within the article.

## Abbreviations

The following abbreviations are used in this manuscript:

DASH	Disabilities of the Arm, Shoulder, and Hand
EE	Eccentric Exercise
NPRS	Numerical Pain Rate Scale
PE	Percutaneous Electrolysis
PNS	Percutaneous peripheral Nerve Stimulation
PPT	Pressure Pain Threshold
ROM	Range of Motion
SPADI	Shoulder Pain and Disability Index
TENS	Transcutaneous Electrical Nerve Stimulation
US	Therapeutic Ultrasound
